# Machine learning to predict the occurrence of thyroid nodules: towards a quantitative approach for judicious utilization of thyroid ultrasonography

**DOI:** 10.3389/fendo.2024.1385836

**Published:** 2024-05-07

**Authors:** Qijun Liang, Zhenhong Qi, Yike Li

**Affiliations:** ^1^ Health Management Center, Foshan Hospital of Traditional Chinese Medicine, Foshan, Guangdong, China; ^2^ Department of Otolaryngology-Head and Neck Surgery, Vanderbilt University Medical Center, Nashville, TN, United States

**Keywords:** machine learning, thyroid nodule, ultrasonography, precision medicine, artificial intelligence, nomograms, logistic models, random forest

## Abstract

**Introduction:**

Ultrasound is instrumental in the early detection of thyroid nodules, which is crucial for appropriate management and favorable outcomes. However, there is a lack of clinical guidelines for the judicious use of thyroid ultrasonography in routine screening. Machine learning (ML) has been increasingly used on big data to predict clinical outcomes. This study aims to leverage the ML approach in assessing the risk of thyroid nodules based on common clinical features.

**Methods:**

Data were sourced from a Chinese cohort undergoing routine physical examinations including thyroid ultrasonography between 2013 and 2023. Models were established to predict the 3-year risk of thyroid nodules based on patients’ baseline characteristics and laboratory tests. Four ML algorithms, including logistic regression, random forest, extreme gradient boosting, and light gradient boosting machine, were trained and tested using fivefold cross-validation. The importance of each feature was measured by the permutation score. A nomogram was established to facilitate risk assessment in the clinical settings.

**Results:**

The final dataset comprised 4,386 eligible subjects. Thyroid nodules were detected in 54.8% (n=2,404) individuals within the 3-year observation period. All ML models significantly outperformed the baseline regression model, successfully predicting the occurrence of thyroid nodules in approximately two-thirds of individuals. Age, high-density lipoprotein, fasting blood glucose and creatinine levels exhibited the highest impact on the outcome in these models. The nomogram showed consistency and validity, providing greater net benefits for clinical decision-making than other strategies.

**Conclusion:**

This study demonstrates the viability of an ML-based approach in predicting the occurrence of thyroid nodules. The findings highlight the potential of ML models in identifying high-risk individuals for personalized screening, thereby guiding the judicious use of ultrasound in this context.

## Introduction

Thyroid nodules are a prevalent condition detectable in up to 67% of individuals (1–4). Although the majority of cases involve benign and asymptomatic lesions that necessitate no intervention, approximately 5-15% of them are malignant or indicative of thyroid diseases, such as hyper- and hypo-thyroidism (5–7). These thyroid conditions, especially cancers, often exhibit a favorable prognosis when identified early. Early detection allows for timely and tailored treatment strategies, thereby increasing the likelihood of a successful outcome (8–10).

Ultrasound has become a widely utilized method for thyroid examination by virtue of its non-invasive nature, fair cost-effectiveness, and broad accessibility. Equipped with linear probes that deliver high-resolution detail of the thyroid gland, ultrasound exhibits a remarkable sensitivity in detecting early-stage lesions as small as a few millimeters (10, 11). Additionally, ultrasound can unveil patterns of vascularity, characterize the nature of the mass, aid in the assessment of adjacent tissues, and offer real-time guidance for biopsy. As a result, thyroid ultrasonography has emerged as the primary tool for thyroid imaging, playing a pivotal role in the global assessment of thyroid diseases (12).

Although broadly regarded as a preferred means of assessing the thyroid, the clinical indication for thyroid ultrasonography varies considerably across the global healthcare system. In some countries like China, thyroid ultrasonography is routinely included in regular health examinations. However, the excessive use of ultrasound may place additional strain on medical resources and substantially increase healthcare costs (13–15). Conversely, in regions where initial thyroid assessment relies primarily on palpation, ultrasound is typically not indicated until a thyroid mass grows into a palpable size or causes symptoms (7, 10). Therefore, a considerable number of thyroid nodules remain unrevealed at early stages, potentially delaying diagnosis and treatment and causing suboptimal outcomes (16, 17). In this regard, the judicious utilization of ultrasound for thyroid examination has gained increasing attention from healthcare professionals (18, 19). Nevertheless, there is still a lack of clinical guidelines providing recommendations for the appropriate circumstances in which ultrasound should be prescribed for screening thyroid nodules.

Machine learning (ML) is a subset of artificial intelligence empowering computers to learn from historical data and predict outcomes for new data based on acquired knowledge. With the advent of the big data era, ML has been increasingly applied to perform predictive modeling in medicine (20–22). ML models exhibit promising performance, often matching or surpassing human judgement across diverse tasks such as disease detection, diagnosis, and risk prediction (23, 24). One notable advantage of ML over traditional statistics is its ability to function effectively with minimal assumptions about data characteristics. This makes ML particularly valuable in situations where data lack a controlled arm or involve intricate nonlinear interactions among predictor variables (25).

The objective of this study is to establish an effective method for assessing the risk of thyroid nodules. The development of thyroid nodules is associated with a mixed combination of biological, lifestyle, and environmental factors, such as age and metabolism (2, 26–28). In this study, electronic health record data, encompassing demographics, anthropometrics, and common laboratory tests, were collected from a large single-center cohort undergoing routine physical examinations including thyroid ultrasonography. ML models were constructed to predict thyroid nodules based on commonly accessible clinical features. The findings from this study not only suggest a clinically feasible approach that can guide the judicious use of thyroid ultrasonography, but also provide insight into the important factors associated with the occurrence of thyroid nodules.

## Materials and methods

### Study cohort

This study was conducted in full accordance with Good Clinical Practice and Declaration of Helsinki. Ethical approval was granted by the Ethics Committees at Foshan Hospital of Traditional Chinese Medicine (document number: KY-2022-151). Data were retrospectively collected from adults who received routine health examinations at the Health Management Center of this tertiary hospital between 2013 and 2023. Thyroid ultrasound, which was included in a comprehensive health examination package designed for early detection of health issues, was performed at patients’ discretion regardless of clinical indications. Individuals were excluded from the analysis if they (1): had a history of known thyroid diseases such as hyperthyroidism, hypothyroidism, subacute thyroiditis, and Hashimoto thyroiditis; or (2) had a history of thyroid therapy, including any medication, surgery, or radiotherapy; or (3) were pregnant or lactating; or (4) had any missing data at baseline. Patients were scheduled to follow up annually after their initial visits and observed until December 31st, 2023.

### Data acquisition

Candidate independent variables included patients’ baseline characteristics (sex, age, body mass index, waist circumference, and mean arterial pressure) and laboratory test results (fasting blood glucose [FBG], triglycerides, total cholesterol, low-density lipoprotein cholesterol [LDL-C], high-density lipoprotein cholesterol [HDL-C], uric acid, alanine transaminase, aspartate aminotransferase, γ-glutamyl transpeptidase, and creatinine). These variables were selected by availability and potential association with the development of thyroid nodules (29, 30). All predictor variables were obtained at baseline during the initial visit.

The dependent variable was the presence or absence of thyroid nodules assessed through ultrasound at each visit. Thyroid nodules were considered present if any discrete lesions within the thyroid gland appeared radiologically distinct from the surrounding parenchyma. These nodules could exhibit solid, spongiform, cystic, or mixed components. Ultrasound examinations were conducted on patients in a supine position by senior sonographers with over 10 years of experience, using a B-mode high-resolution tomographic ultrasound system (Esaote, Genova, Italy). All images were reviewed by at least one independent clinical expert before final reports were generated.

### Data preprocessing

Logarithmic transformation was applied to continuous variables that exhibited skewed distribution. Time to event was coded as the number of years between the initial visit and the onset of thyroid nodules or the last follow-up visit, whichever occurred earlier. The ground truth label was determined based on the presence or absence of thyroid nodules by the end of a 3-year observation period. Subjects exhibiting thyroid nodules at baseline or in less than 3 years from the initial visit were labeled as nodule-positive, while those who remained disease-free or only exhibited nodules after 3 years were labeled as nodule-negative. Individuals who were lost to follow-up or had missing ultrasound data were discarded. A fivefold cross-validation method was applied to train and test the ML models. Specifically, the dataset was split randomly into a training set (80%) and a test set (20%). This process was repeated 5 times, resulting in 5 distinct test sets. During training, a random selection of 20% data from the training set was employed for model validation. Data were split in a stratified fashion to ensure consistent class distribution in each subset as the entire dataset.

### Model development

Models underwent training and testing in a binary classification task to assess the 3-year risk of thyroid nodules based on baseline features. Four ML algorithms were employed, including logistic regression, random forest, extreme gradient boosting (XGBoost), and light gradient boosting machine (LightGBM). Specifically, random forest is a classic ensemble method that combines the outputs of multiple parallel decision trees for making predictions (31). XGBoost comprises a group of decision trees that are weak prediction learners connected in a sequential fashion. This gradient boosting structure allows a new learner to concentrate on areas where the existing learners are performing poorly, thereby reducing the error in the entire model. The LightGBM has a similar structure to the XGBoost but uses a different strategy to split the data. These ML models represent the state-of-the-art ML techniques that show remarkable outcomes in a variety of tasks (32–35). Logistic regression served as a baseline model to allow unbiased performance assessment for these ML models.

A grid search was performed to determine the optimal hyperparameters for each algorithm. Each ML algorithm with the best hyperparameters was trained to achieve convergence on the training set. The cutoff threshold of each model was determined at the top left corner of the receiver operating characteristic curve (i.e., the maximum sum of sensitivity and specificity) from the validation set and applied unchangeably to the test set. All training and testing sessions were done in a Python 3.8 environment using scikit-learn v1.02, an open-source package for ML.

### Feature importance

The importance of each predictor variable in a model was quantified by the permutation score on the test set. This score is defined as the decrease in model performance [measured by the area under the receiver operating characteristic curve (AUROC)] when all values of a given variable are randomly shuffled. Essentially, this procedure breaks the relationship between the feature and the outcome, and the extent of performance reduction indicates the reliance of the model on that particular feature. This process was iterated 50 times for each variable in a model to obtain an average score.

### Statistical analysis

Descriptive statistics were applied to characterize the baseline features of this cohort. The performance of each ML model was evaluated based on a range of metrics, including accuracy, recall, specificity, precision, F1 score, AUROC, and area under the precision-recall curve. Results were averaged over 5 cross-validation folds and are presented as means with 95% confidence intervals. Cochrane’s Q test was employed to evaluate differences in predictive performance among the models, with statistical significance determined at an alpha threshold of 0.05.

To facilitate clinical application, a nomogram visually depicting the risk of thyroid nodules was developed using the simpleNomo package in Python (36). A calibration curve was utilized to measure the consistency between the predicted risks and the actual outcomes. The clinical benefit of the nomogram was evaluated by decision curve analysis (37, 38). All statistical analyses were conducted using Python 3.8 and Excel (Microsoft Corporation, Redmond, WA).

## Results

### Cohort characteristics

**Figure 1:**
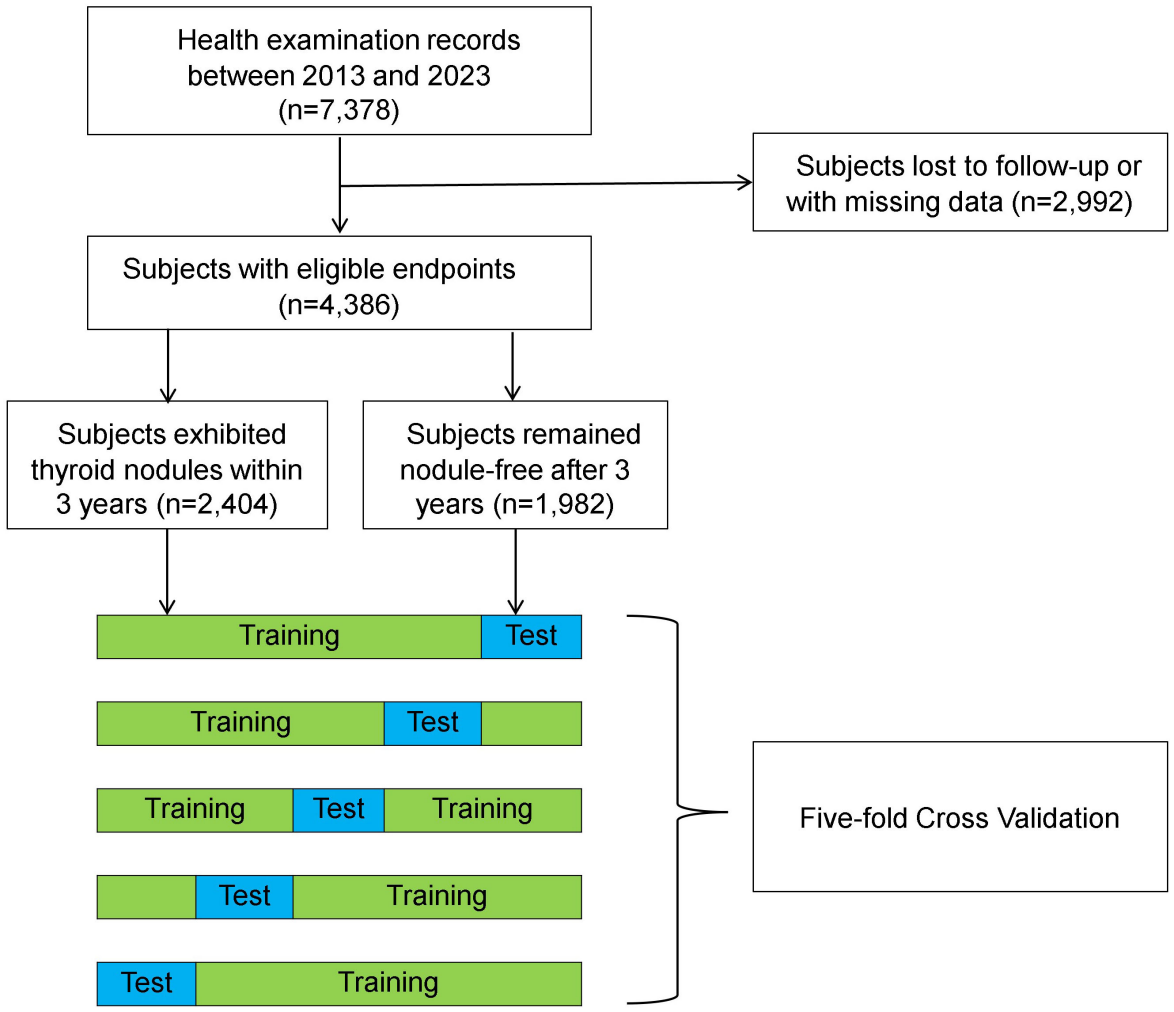
A flow chart outlining the study design.

The final dataset comprised 4,386 individuals who met the inclusion criteria for the study (**Figure 1**). Subjects were predominantly male (58.8%) and generally in middle age (38.2 years) during their initial visits (**Table 1**). A total of 54.8% individuals (n = 2,404) exhibited thyroid nodules, either at baseline (n = 1,841) or within the 3-year observation period (n = 563).

**Table 1:** Baseline characteristics of the dataset.

Variables	Statistics
Sex (Male: Female)	2578: 1808
Age (years)	38.2 ± 10.8
BMI (kg/m2)	22.9 (20.7, 25.3)
WC (cm)	83.0 ± 7.5
MAP (mmHg)	88.7 (82.0, 95.3)
FBG (mmol/L)	5.3 (4.9, 5.7)
TG (mmol/L)	1.1 (0.7, 1.7)
TCH (mmol/L)	4.9 (4.3, 5.6)
LDL-C (mmol/L)	2.9 (2.4, 3.4)
HDL-C (mmol/L)	1.4 (1.3, 1.6)
UA (μmol/L)	359.3 (295.6, 427.9)
ALT (U/L)	22.3 (16.4, 32.1)
AST (U/L)	20.9 (17.3, 25.4)
GGT (U/L)	22.3 (16.5, 33.6)
Cr (μmol/L)	69.5 (59.0, 79.8)

Results are presented as mean ± standard deviation or median (first quantile, third quantile). BMI, Body Mass Index; WC, Waist Circumference; MAP, Mean Arterial Pressure; FBG, Fasting Blood Glucose; TG, Triglyceride; TCH, Total Cholesterol; LDL-C, Low-Density Lipoprotein Cholesterol; HDL, High-Density Lipoprotein Cholesterol; UA, Uric Acid; ALT, Alanine Transaminase; AST, Aspartate Aminotransferase; GGT, γ-Glutamyl Transpeptidase; Cr, Creatinine.

### Model performance

The optimal hyperparameters for each algorithm are presented in **Table 2**. Overall, these models successfully predicted the outcome in approximately two-thirds of individuals (**Table 3**). All ML models demonstrated superior performance compared to the baseline logistic regression model (p<0.001). Despite a modest difference in overall predictability, each model revealed similar recall and specificity scores, suggesting a balanced performance in identifying patients with and without thyroid nodules.

**Table 2:** Best hyperparameters settings for each model.

Algorithms	Hyperparameters
LR	penalty=‘l2’, class_weight = ‘balanced’, max_iter = 100000, C = 8, solver = ‘liblinear’
RF	criterion=‘gini’, max_depth = 7, max_features = 6, min_samples_leaf = 60, min_samples_split = 140, n_estimators = 32
LightGBM	subsample=0.5, objective = ‘binary’, learning_rate = 0.1, gamma = 0.1, ‘colsample_bytree’: 0.7, ‘min_child_samples’: 100, ‘n_estimators’: 64, ‘num_leaves’: 8
XGBoost	colsample_bytree’: 0.6, ‘max_depth’: 4, ‘min_child_weight’: 25, ‘n_estimators’: 64, learning_rate = 0.1, subsample=0.5, gamma = 0.1, objective = ‘binary:logistic

LR, Logistic Regression; RF, Random Forest; LightGBM, Light Gradient Boosting Machine; XGBoost, Extreme Gradient Boosting.

**Table 3:** Performance of each model in predicting the 3-year onset of thyroid nodules.

Models	Accuracy	Recall	Specificity	Precision	AUC	AP	F1 Score	*p*
LR	0.65 [0.63, 0.67]	0.60 [0.51, 0.70]	0.71 [0.62, 0.80]	0.72 [0.69, 0.76]	0.72 [0.71, 0.72]	0.76 [0.76, 0.77]	0.65 [0.60, 0.70]	
RF	0.68 [0.67, 0.69]	0.66 [0.64, 0.68]	0.71 [0.66, 0.76]	0.74 [0.70, 0.77]	0.75 [0.74, 0.77]	0.78 [0.76, 0.81]	0.69 [0.69, 0.70]	<0.001
LightGBM	0.69 [0.67, 0.70]	0.66 [0.62, 0.70]	0.72 [0.68, 0.76]	0.74 [0.73, 0.76]	0.76 [0.74, 0.77]	0.79 [0.77, 0.81]	0.70 [0.68, 0.72]	<0.001
XGBoost	0.68 [0.67, 0.70]	0.63 [0.57, 0.69]	0.74 [0.70, 0.78]	0.75 [0.73, 0.76]	0.76 [0.74, 0.77]	0.79 [0.78, 0.81]	0.68 [0.65, 0.72]	<0.001

All outcomes are averaged over five rounds of cross-validation and presented as mean [95% confidence interval]. LR, Logistic Regression; RF, Random Forest; LightGBM, Light Gradient Boosting Machine; XGBoost, Extreme Gradient Boosting; AUC, Area Under the Receiver Operating Characteristic Curve; AP, Average Precision (i.e., Area Under the Precision-Recall Curve).

### Critical predictors

**Figure 2:**
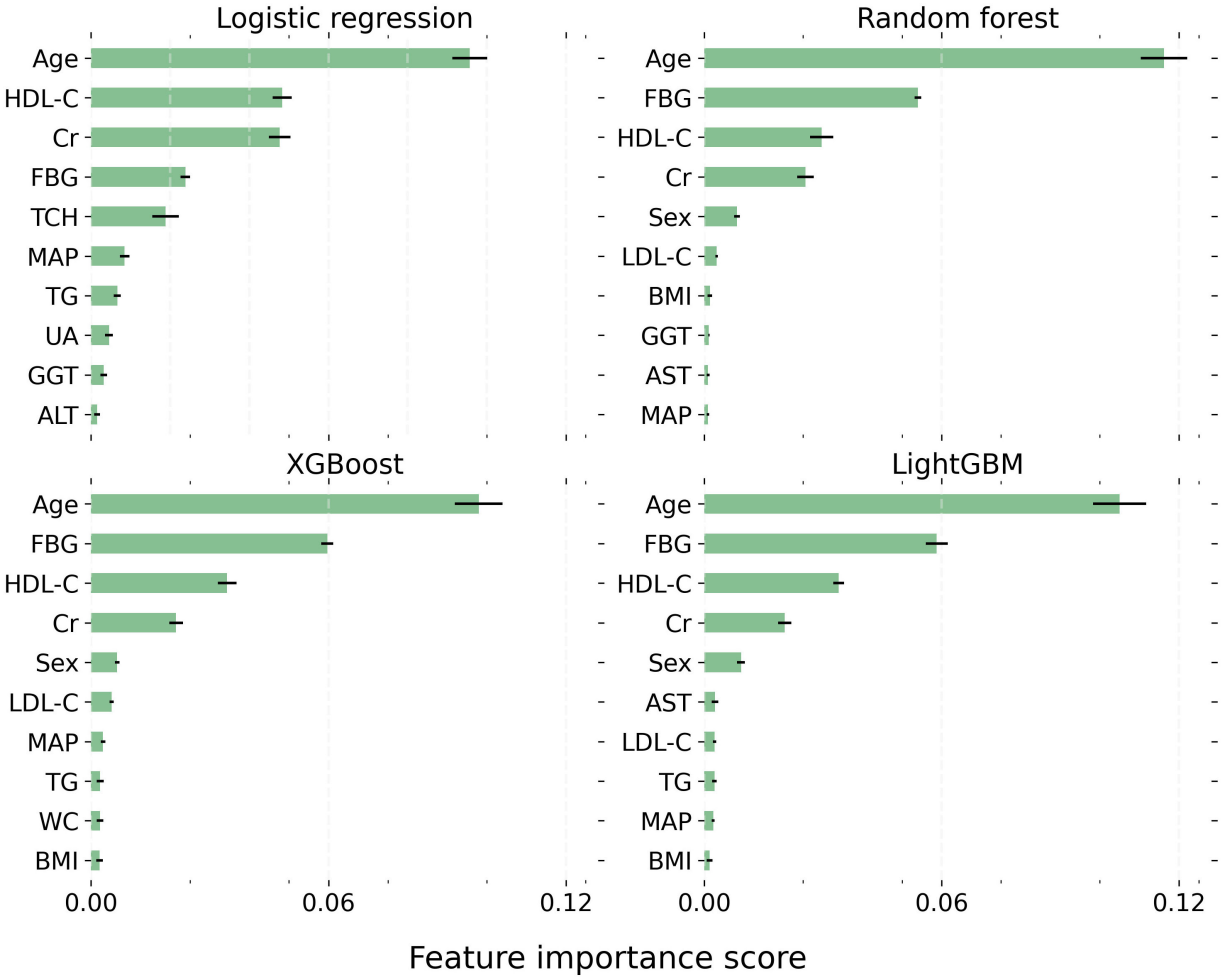
The top 10 most critical features for each model. Each bar represents the mean importance score, with the black horizontal line indicating the standard error of the mean. LightGBM, Light Gradient Boosting Machine; XGBoost, Extreme Gradient Boosting. BMI, Body Mass Index; WC, Waist Circumference; MAP, Mean Arterial Pressure; FBG, Fasting Blood Glucose; TG, Triglyceride; TCH, Total Cholesterol; LDL-C, Low-Density Lipoprotein Cholesterol; HDL, High-Density Lipoprotein Cholesterol; UA, Uric Acid; ALT, Alanine Transaminase; AST, Aspartate Aminotransferase; GGT, γ-Glutamyl Transpeptidase; Cr, Creatinine.

The top 10 critical features influencing the development of thyroid nodules were largely consistent across all models (**Figure 2**). These pivotal factors encompassed age, HDL-C, FBG, creatinine, LDL-C, triglycerides, sex, and mean arterial pressure. Notably, age exhibited the most substantial impact on the outcome, maintaining the highest rank in every model. HDL-C, creatinine, and FBG were also identified as significant predictors, consistently appearing among the top four positions in all four models.

### Nomogram

**Figure 3:**
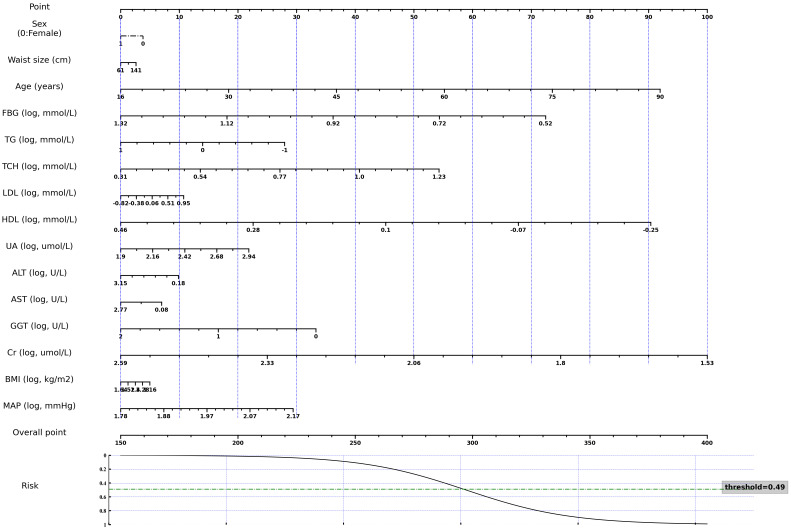
A nomogram for estimating the 3-year risk of thyroid nodule. Variables marked with “log” require logarithmic transformation with a base of 10 to obtain the proper scores. FBG, Fasting Blood Glucose; TG, Triglyceride; TCH, Total Cholesterol; LDL, Low-Density Lipoprotein Cholesterol; HDL, High-Density Lipoprotein Cholesterol; UA, Uric Acid; ALT, Alanine Transaminase; AST, Aspartate Aminotransferase; GGT, γ-Glutamyl Transpeptidase; Cr, Creatinine; BMI, Body Mass Index; MAP, Mean Arterial Pressure. This nomogram, along with other pre-trained models and code, are publicly accessible at the following repository: https://github.com/huntlylee/Thyroid-nodule.

**Figure 4:**
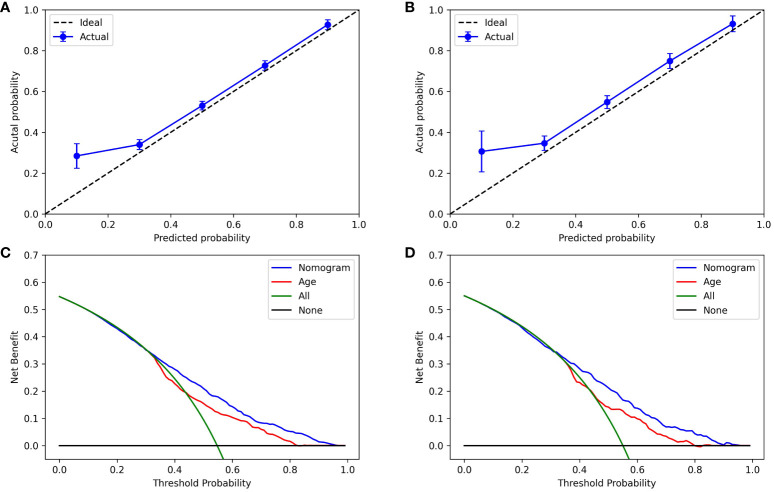
Calibration plots and decision curve analysis of the nomogram. Calibration plots measure the accuracy of the nomogram’s predictions by comparing the average predicted risks against the actual observed probabilities, employing a bootstrapping technique in both the training **(A)** and validation **(B)** sets. The decision curve analysis quantifies the trade-off between the risk of taking unnecessary actions (i.e. unwarranted thyroid ultrasounds) and the advantages of appropriate interventions across various threshold levels for each assessment method in the training **(C)** and validation **(D)** cohorts.

The nomogram was derived from the logistic regression model incorporating all features to optimize predictability (**Figure 3**). It demonstrated comparable performance in predicting the occurrence of thyroid nodules in both the training and validation sets, with accuracy scores of 0.67 and 0.66, and AUROC scores of 0.72 and 0.72, respectively. The calibration curve indicated a good agreement between the nomogram’s predicted probabilities and the actual observations. Furthermore, the decision curve analysis revealed that the nomogram offered greater net benefits in the evaluation of thyroid nodules compared to strategies that rely solely on age or use an all-or-none approach, especially at a probability threshold above 0.35 (**Figure 4**). Additionally, an Excel spreadsheet has been provided to facilitate the clinical implementation of this model on electronic devices for assessing the risk of thyroid nodules (**Supplementary Material**).

## Discussion

Evaluating the risk of thyroid nodule onset holds significant benefits for tailoring monitoring strategies and guiding the appropriate use of ultrasound in this context. This study demonstrated the feasibility of utilizing ML to forecast the 3-year risk of thyroid nodules based on readily accessible baseline features. All models were able to predict the occurrence of thyroid nodules in approximately two-thirds of individuals, displaying a sensitivity of up to 66%. This performance suggests the clinical potential as a regular screening tool for thyroid nodules in the general population. In contrast to recent ML studies that primarily focused on the detection, classification, or prognosis of thyroid malignancies (39–42), this study concentrated on the onset of thyroid nodules — a more common clinical task that is upstream to all these later-stage models and of public health significance. The current dataset also represents a sizable cohort with ample observation time and reliable study endpoints. Employing a fivefold cross-validation approach and a comprehensive set of performance metrics allows unbiased evaluation of the ML models. Quantifying the feature importance also provides insightful findings regarding thyroid nodule pathogenesis for future mechanistic research. This study reinforces the potential of artificial intelligence to revolutionize healthcare in the era of big data. With its capacity to generate timely and reliable predictions in intricate tasks, ML is poised to become an integral part of routine clinical practice, notably advancing personalized medicine.

Although ultrasound has been proven as a cost-effective approach for thyroid assessment, it is not routinely prescribed during heath checkup in many countries. Instead, thyroid is primarily evaluated through physical examinations, with further assessments being determined by physicians’ judgments. Palpation through fingers relies on clinicians’ experiences and skills, which results in inter-operator variability and suboptimal sensitivity (3, 43, 44). Consequently, most thyroid nodules may only be identified after progressing into palpable sizes or causing perceivable symptoms, leading to a potential delay in diagnosis and treatment of the underlying conditions. Although certain risk factors, such as age, obesity, and smoking, are known to be associated with the occurrence of thyroid nodules, there is currently no specific guideline that offers instruction for screening based on these factors. This study addresses this gap by the development of risk stratification models. These quantitative models demonstrate favorable performance in forecasting the 3-year risk of thyroid nodules based on common clinical features, suggesting an evidence-based approach for clinical decision-making that is deemed less biased compared to the subjective judgments. For one thing, these ML models may aid in estimating the need for further thyroid assessment during routine health examination. A timely ultrasound is expected to allow detection of tiny nodules before they increase in size or exhibit symptoms, potentially facilitating the early diagnosis and treatment of thyroid diseases and improving outcomes. For another thing, these models can also effectively spare low-risk individuals from unnecessary assessments, thus avoiding overtreatment or excess health spending. In China, with an annual estimate of 495 million individuals undergoing routine physical examination and a detection rate of 20% for thyroid nodules (45–47), these models are anticipated to substantially reduce thyroid ultrasonography by at least 285 million cases and save 4 billion dollars in costs per year.

The clinical viability of this quantitative approach is supported by the commendable model performance in identifying both nodule-positive and -negative patients, in addition to the net benefits of the nomogram over alternative strategies. These models can be developed into applications or integrated into electronic medical record systems for rapid risk assessment and clinical triage. In this study, all pre-trained models, along with a detailed instruction manual, have been shared in a public repository (https://github.com/huntlylee/Thyroid-nodule), allowing straightforward inference on user-provided data through simple command-line inputs. To aid in the clinical adoption of this model, a nomogram and a user-friendly spreadsheet have been provided, designed to support physicians across a range of ML expertise.

Identifying factors associated with disease onset is essential before considering focused treatment or preventive strategies. In line with prior studies, this research also identifies several patient characteristics, such as age, HDL-C, and FBG, as being associated with the development of thyroid nodules. Firstly, age is widely recognized as a significant risk factor for thyroid nodule (29, 48), potentially due to age-related oxidative stress and the involvement of vascular endothelial growth factor (49, 50). Evidence suggests that older adults are more likely to develop thyroid malignancies of high-risk histology, highlighting the need of early detection of thyroid nodules (26). Secondly, there is a noted prevalence of thyroid nodules in individuals with metabolic disorders like diabetes and hyperlipidemia (51, 52), which is corroborated by the identification of FBG, LDL-C, HDL-C, and triglycerides as critical predictors in this study. The prevailing theory suggests that metabolic disorders could promote thyroid cell growth through interactions between insulin and thyroid stimulating hormone (53, 54). Metabolic disorders might also trigger oxidative stress, causing cellular damage and affecting genomic stability in the thyroid (49, 50, 55–57). Additionally, creatinine levels have been found to be associated with thyroid nodules (29, 58), although the cause remains unclear. Creatinine might also act as a proxy marker for sex or other risk factors that exhibit sex discrepancies. Further research is required to elucidate the underlying mechanisms of these factors in the pathogenesis of thyroid nodules.

While previous studies have yielded inconclusive findings regarding the advantage of ML over traditional statistics in performing different clinical tasks (34, 35, 59, 60), it is generally believed that complex ML models often require big data to achieve optimal performance (61). This study involved a substantial dataset of more than 4,000 subjects with a balanced class distribution, where all three ML models showed significant improvements over the standard logistic regression, albeit to a modest extent. This finding reinforces the potential of ML in predicting medical or epidemiological outcomes when large datasets are available. Nonetheless, classic regression approaches may continue to play a pivotal role in these tasks by virtue of the model simplicity, which can mitigate bias and overfitting in scenarios with smaller or imbalanced datasets (35, 62). Moreover, the superior explainability of simple regression models over complex ML algorithms may make them more suitable for predicting clinical outcomes, as explainable equations may facilitate clinical adaptation. In this study, the logistic regression model was converted into a nomogram and a simple formula that can be implemented in an Excel spreadsheet, enabling effective utilization of this method by clinicians without the need for ML expertise.

Several limitations should be acknowledged in this study. Firstly, only a monocentric dataset was obtained. While this dataset comprises a substantial cohort with reliable ground truths, it may still be susceptible to certain biases related to race, region and sex. Notably, there was a minor gender disparity within the cohort, which may be attributed to a higher exclusion rate of female participants who are generally more susceptible to thyroid conditions. Hence, the generalizability of these models might necessitate further validation on a wider patient population. Secondly, only a limited number of variables were employed for prediction. Although these variables were chosen for their relevance and data availability, there are other risk factors not currently accessible in the database, such as smoking, family history, and radiation exposure. Including these features is likely to enhance the model performance. Thirdly, only a handful of models were tested in this early feasibility study, although they are representative of cutting-edge ML techniques. Given the rapid evolution in both medical research and data science, future studies will likely assess new ML approaches as they become available. Lastly, the critical features identified may indicate associations rather than causation due to the study’s retrospective nature. Although they offer insights for further investigation into disease pathogenesis, it will be essential to conduct mechanistic and prospective studies to understand the causal relationships and their roles in the development of thyroid nodules.

Future research should aim to address these limitations and facilitate model deployment in clinical settings. For example, additional variables linked to the onset of thyroid nodules will be collected to improve model performance. A broader dataset will be compiled from multiple independent hospitals to evaluate and enhance the generalizability of these models. This approach may also be extended to forecast other clinically significant outcomes, such as the trajectory, malignancy, or prognosis of thyroid nodules. These ML models will be integrated into existing electronic health record systems with user-friendly interfaces to facilitate human-machine interaction and enable efficient decision-making. Efforts are underway to collect more data and test these models in prospective studies. The ultimate objective of this research line is to establish a robust artificial intelligence system that can effectively support clinicians in the evaluation and management of thyroid diseases.

## Conclusion

In conclusion, this study showed the feasibility of ML in predicting the occurrence of thyroid nodules. Age, HDL-C, FBG, and creatinine levels were identified as the critical factors associated with the outcome. These findings pave the way for a quantitative approach in guiding the judicious use of ultrasound for personalized screening. Future research will involve conducting external validation and enhancing the model by incorporating additional predictor variables.

## Data availability statement

The raw data supporting the conclusions of this article will be made available by the authors, without undue reservation. All models and code are available via: https://github.com/huntlylee/Thyroid-nodule.

## Ethics statement

The studies involving humans were approved by the Ethics Committees at Foshan Hospital of Traditional Chinese Medicine. The studies were conducted in accordance with the local legislation and institutional requirements. Written informed consent for participation was not required from the participants or the participants’ legal guardians/next of kin in accordance with the national legislation and institutional requirements.

## Author contributions

QL: Conceptualization, Data curation, Funding acquisition, Investigation, Visualization, Writing – original draft. ZQ: Project administration, Resources, Writing – review & editing. YL: Conceptualization, Data curation, Formal analysis, Methodology, Software, Supervision, Validation, Visualization, Writing – original draft, Writing – review & editing.
